# Multifaceted intervention to increase the delivery of alcohol brief interventions in primary care: a mixed-methods process analysis

**DOI:** 10.3399/BJGP.2022.0613

**Published:** 2023-09-05

**Authors:** Elizabeth Sturgiss, Jenny Advocat, Tina Lam, Suzanne Nielsen, Lauren Ball, Nilakshi Gunatillaka, Catherine Martin, Chris Barton, Chun Wah Michael Tam, Helen Skouteris, Danielle Mazza, Grant Russell

**Affiliations:** School of Primary and Allied Health Care, Monash University, Melbourne, Australia.; School of Primary and Allied Health Care, Monash University, Melbourne, Australia.; Monash Addiction Research Centre, Eastern Health Clinical School, Monash University, Frankston, Victoria, Australia.; Monash Addiction Research Centre, Eastern Health Clinical School, Monash University, Frankston, Victoria, Australia.; Grad Dip Health Economics and Health Policy, chair of community health and wellbeing, University of Queensland, Brisbane, Australia; Menzies Health Institute Queensland and School of Health Sciences and Social Work, Griffith University, Brisbane, Australia.; School of Primary and Allied Health Care, Monash University, Melbourne, Australia.; School of Public Health and Preventive Medicine, Monash University, Melbourne, Australia.; GAICD, CF, head;; Primary and Integrated Care Unit, South Western Sydney Local Health District, Liverpool, New South Wales, Australia; conjoint senior lecturer, School of Population Health, University of New South Wales, Sydney, New South Wales, Australia.; Health and Social Care Unit, School of Public Health and Preventive Medicine, Monash University, Clayton, Victoria, Australia; Warwick Business School, University of Warwick, Coventry, UK.; GAICD, CF, head;; Department of General Practice, School of Public Health and Preventive Medicine, Monash University, Melbourne, Australia.

**Keywords:** alcohol drinking, brief interventions, COVID-19, implementation science, primary health care

## Abstract

**Background:**

Brief interventions (BIs) are effective for reducing harmful alcohol consumption, but their use in primary care is less frequent than clinically indicated. The REducing AlCohol- related Harm (REACH) project aimed to increase the delivery of BIs in primary care.

**Aim:**

To assess the effectiveness of the REACH programme in increasing alcohol BIs in general practice and explore the implementation factors that improve or reduce uptake by clinicians.

**Design and setting:**

This article reports on a sequential, explanatory mixed-methods study of the implementation of the REACH project in six general practice clinics serving low-income communities in Melbourne, Australia.

**Method:**

Time-series analyses were conducted using routinely collected patient records and semi-structured interviews, guided by the consolidated framework for implementation research.

**Results:**

The six intervention sites significantly increased their rate of recorded alcohol status (56.7% to 60.4%), whereas there was no significant change in the non-intervention practices (344 sites, 55.2% to 56.4%).

**Conclusion:**

REACH resources were seen as useful and acceptable by clinicians and staff. National policies that support the involvement of primary care in alcohol harm reduction helped promote ongoing intervention sustainability.

## INTRODUCTION

Alcohol is Australia’s most harmful drug with the highest per capita death rate reported in 2021 in a decade (5.4 age- standardised deaths per 100 000) and alcohol use accounting for 4.5% of the total disease and injury burden.^1^ Alcohol use increased during the COVID-19 pandemic in a number of countries.^1^^–^^3^ In the US, alcohol- related deaths increased more than 20%,^4^ highlighting the need for innovative solutions to reduce alcohol-related harms. There is a complex relationship between socioeconomic disadvantage, alcohol consumption, and alcohol- related harms.^5^ International evidence consistently demonstrates that people in socioeconomically disadvantaged groups experience higher levels of alcohol-related harm than those in advantaged groups with similar amounts of alcohol exposure.^5^

The World Health Organization (WHO) recommends alcohol brief interventions (ABIs) be delivered in primary care.^6^ ABIs involve assessing the amount of alcohol a person consumes and then offering individualised advice and support to reduce the associated health risks. The delivery of ABIs in primary care is associated with a reduction of approximately 20 g of alcohol (two standard drinks) per week at 1 year.^7^ Australia’s National Alcohol Strategy also recognises the key role of primary care in screening and ABIs.^8^ The Royal Australian College of General Practitioners (RACGP) recommends universal screening with all individuals aged ≥15 years asked about alcohol use every 2–4 years.^9^ Despite this, ABIs in Australian primary care are not delivered as often as clinically indicated,^10^ with only 56.2% of regular patients aged ≥15 years having an alcohol status recorded in their general practice electronic record.^11^

**Table:** How this fits in

Brief interventions can reduce alcohol- related harm when delivered in general practice, but there is an implementation gap in routine clinical practice. The REACH programme, which includes resources for patients, clinicians, and clinics, can improve alcohol recording in the general practice setting. Enhanced alignment between national policy and clinical need can support preventive health innovations through existing channels. When appropriately resourced and supported, general practice can deliver alcohol brief interventions in daily practice.

Worldwide, there have been other programmes that focus on improving the delivery of ABIs. Most recently WHO Europe has released a toolkit for an integrated approach to prevention of chronic health conditions.^12^ Most successful strategies for increasing ABIs rely on financial incentives,^13^ but a recent interrupted time-series analysis from the UK lends caution with no increase in alcohol screening with the introduction of incentives, but the reduction of alcohol screening when the incentives were withdrawn from real-world settings.^14^ UK researchers have emphasised the need for more comprehensive strategies for ABI implementation.^15^

As the first point of contact with the healthcare system, primary care is a critical setting for the delivery of preventive health care and is defined by its aim to provide whole-person, longitudinal care.^16^ The Australian Government subsidises universal health care for primary care through ‘Medicare’. Most primary care services are provided by GPs and practice nurses in privately owned community-based clinics. Across Australia, primary health networks (PHNs) are Australian Government-funded organisations providing coordination and support to general practices and commissioning services based on local unmet needs. They are similar in function to other primary care commissioning bodies in the UK and Canada^17^ and have key performance indicators that are set by the Australian Government.^18^ Most PHNs employ staff to liaise between the PHN and general practices, and provide practices with individualised support and feedback. PHN key performance indicators are set by the Australian Government and are based on the seven national priority areas including mental health, Aboriginal and Torres Strait Islander health, population health, workforce, digital health, aged care, and alcohol and other drugs.^19^

To address this known gap between evidence and practice, the overall objective of the REducing AlCohol-related Harm (REACH) project was to increase the use of ABIs in general practice. The authors of the current study focused on developing an approach that was acceptable and feasible for low-income groups by using principles of equity in intervention development.^20^

The aim of this article was to assess the effectiveness of the REACH programme in increasing ABIs in general practice and explore the implementation factors that improve or reduce uptake by clinicians.

## METHOD

This was a single-arm, implementation trial with an observational control group using a sequential, explanatory mixed- methods evaluation.^21^ The research questions and methods were published a priori.^22^ As a result of the restrictions of the COVID-19 pandemic, all interviews had to be conducted remotely and the REACH resources were made available online; more detail is found below in ‘Implementation strategy’.

The study was conducted between January 2020 and March 2021 in low- income communities in Melbourne, Australia’s second most populated city. During this time, the Melbourne region experienced significant COVID-19 cases and was subject to lockdown restrictions. Many general practice clinics rapidly transitioned to include telehealth,^23^ and were later heavily involved in vaccine administration.^24^ General practices were operating under highly stressful and unusual circumstances.^25^

A convenience sampling method was used in this study with the local PHN partner seeking expressions of interest through newsletters and email alerts. Eligibility criteria included: practices in lower socioeconomic areas with at least one clinician willing to use the resources with patients. The PHN passed on contact details of interested practices to the research team.

### Intervention

REACH resources were locally designed in partnership with Enliven Victoria, a health promotion charity, and were directed to patients (waiting room posters and survey, pamphlets, and prompt signs), and clinicians (flowchart, standard drinks chart, and podcast) (full details in Supplementary Box S1). Enliven, at the time of the study, was part of the Victorian network of primary care partnerships, which brought together health and community services organisations to improve health and social wellbeing for vulnerable groups (https://vicpcp.org.au). The resources were based on three pre-existing educational materials.^26^^–^^29^ In the current study the authors aimed for a readability level of school grade 5 or 6 (Flesch– Kincaid) to improve readability for people with low literacy levels, and incorporated simple images depicting diverse individuals. Materials were reviewed for acceptability with consumer representatives. Professional services translated the brochures into the two most common languages in north-west Melbourne other than English — Arabic and Chinese.^30^

### Implementation strategy

The implementation strategy^31^ was guided by normalisation process theory (NPT)^32^ and priming^33^ (Box 1 and Supplementary Box S2). Practices were given written instructions about how to use the resources and were invited to contact the researchers with any questions. This low-intensity approach was intentional to align with possible future scale-up. The PHN’s relationship manager contacted the practice every 3 months to discuss the practice’s data on patient alcohol status and to ask about resource use.

**Box 1.** Implementation strategy^31^ for the REACH project guided by NPT and priming; telehealth adaptation included

**Intervention**	**NPT coherence**	**NPT cognitive participation**	**NPT collective action**	**NPT reflexive monitoring**	**Priming**
**Actors involved**	Practice champion GPs or nurses in practice	PHN Practice champion GPs or nurses in practice	Practice champion GPs or nurses in practice	Practice champion GPs or nurses in practice	Patients
**Action**	Waiting room poster Resources for GPs/nurses Podcast with GPs with AOD expertise Telehealth adaptation	PHN — practice champion relationship Waiting room poster Reflection on data	Waiting room survey Within-consultation resources Pamphlets Telehealth adaptation	PHN and practice meetings: data presentation and discussion	Waiting room resources Consultation room resources Telehealth adaptation
**When the action happens**	Posters distributed at the start of the intervention Resources for GPs or practice nurses available throughout	PHN to engage the practice champion at the start of the intervention PHN to provide feedback every 3 months	All resources provided at the start of the intervention and available throughout	PHN to provide feedback every 3 months	All resources provided at the start of the intervention and available throughout
**Target mechanism triggered by the action**	Encourage discussion between GPs/practice nurses about alcohol BIs to improve understanding Increase GPs/practice nurses’ knowledge and self-efficacy	Relationship building between the PHN and practice via the practice champion Encourage conversations and community building between GPs, practice nurses, and staff in the practice	Resources support and trigger alcohol BIs within consultations SMS/email direct send to patient^a^ Links within electronic medical record^a^	GPs/nurses reflect on how they provide BIs GPs/nurses learn from each other about strategies for alcohol BIs in consultations	‘Primes’ the patient and increases awareness that GPs can talk to you about alcohol Options: SMS from appointment booking system^a^ Practice website links and image^a^ Telehealth ‘waiting room’^a^
**Outcome affected**	Increase practitioner uptake of ABIs in general practice				

a *Telehealth adaptations to the implementation strategy due to the pandemic; see Supplementary Box S2 for full details. ABI = alcohol brief intervention. AOD = alcohol and other drug. BI = brief intervention. NPT = normalisation process theory. PHN = primary health network. REACH = REducing AlCohol-related Harm. SMS = short message service.*

As telehealth consultations increased, the REACH resources were made available online via a commercial online portal that allows clinicians to send the REACH resources directly by email or short message service (SMS) to their patient (Box 1 and Supplementary Box S2). To be able to send emails and SMSs to their patients, practices needed to meet with the portal team (external to the research team) and set up a login process. The SMS costs for sending any materials were covered by the research funding.

### Patient alcohol status data

Across Australia, most general practices use software that records alcohol histories using the AUDIT-C. The PHN then uses the extraction tool Pen CS CAT4^34^ to convert this AUDIT-C data from the electronic medical record into an alcohol status of ‘drinker’, ‘non-drinker’, or it is left blank if no alcohol history is recorded. A blank alcohol history in the patient’s record was taken as a proxy indicator that the general practice team had not previously spoken to the patient about alcohol.^35^ This use of routinely collected data is similar to other studies of primary care where routinely collected data is used,^14^^,^^15^ but in the current study there was not access to individual patient records to cross-check billing items and consultation details.^13^^,^^15^ Without access to medical records, routinely collected data about alcohol screening by PHNs that did not include patient characteristics were used.

The practice incentive program quality improvement (PIPQI) incentive commenced on 1 August 2019.^11^ To receive the PIPQI payment, practices provide data to their PHN as well as complete quality improvement activities. Alcohol recording is captured in the PIPQI. The data were recorded as the number of patient records with a drinking status recorded, not applicable, or not available. Up to 385 non- intervention practices that routinely provide data to the PHN were used as observational practices to compare alcohol recording rates with the REACH practices (average 344 practices each month, range 194–382).

### Statistical analyses

An interrupted time-series approach was used to analyse the time series using generalised linear regression modelling, with a Poisson probability distribution and a log link function.^36^ A heteroskedastic and autocorrelation consistent variance estimator was used, with Newey‒West standard errors. To consider the different total population numbers in the intervention and control practices, the count per month was weighted by the number of patients per month, with the resulting outcome being a rate per month. The outcome was multiplied by 100 to give rates as a percentage per month. The presence of seasonality was examined and did not appear to be present. Outliers were present so an indicator variable representing the outliers was included in the model. The adequacy of the model was investigated by examining the plot of observed model-estimated monthly event count against the observed event count. To determine if a change in alcohol consumption documentation occurred in the intervention group, slopes pre-and post- intervention were compared, with a slope ratio >1 suggesting an increase in the rate of alcohol consumption documentation post-intervention compared with pre- intervention. To determine if the change in the intervention group (pre/post) behaved differently from the control group, an interaction term between group and treatment period was included in the model, with a slope ratio >1 suggesting an increase in the rate of alcohol consumption documentation post-intervention compared with pre-intervention in the intervention group compared with the control group. Statistical significance was defined as a two-sided *P*-value ≤0.05. Stata Statistical Software: release 16 (College Station, TX: StataCorp LP) was the statistical software used for all analyses.

### Interviews

Participants from each practice (GPs, practice managers, and practice nurses) plus the PHN relationship managers were invited to participate in an in-depth, semi- structured interview conducted via Zoom or telephone near the end of the REACH project. Interviews, conducted from May to August 2021, were guided by a semi- structured interview guide. Interviews ranged in length from 18–60 min, with most approximately 30 min. Interviews focused on the REACH resources, processes of implementation, and the relationship between the PHN and practice (see Supplementary Information S1). Interviews were audiorecorded and transcribed. Thematic analysis drew from constructs from NPT^32^ and the consolidated framework for implementation research (CFIR)^37^ plus inductive coding. The CFIR provided a framework to analyse implementation factors across the inner and outer context of the practices including relationships beyond the practices with the PHN and the national policy environment. NPT provided focus on the internal workings of the practice, highlighting barriers and facilitators to implementation (see Supplementary Box S2). Together the use of these complementary frameworks led to the development of four themes that summarise REACH implementation factors.

Interviews were conducted with all PHN staff involved in the REACH project who were still employed at the PHN. All staff and clinicians of the practices who were involved with REACH were invited to participate. Data collection concluded when the authors had interviewed participants from each practice and had sufficient data to explain the process of implementation. Interview guides were designed by the first, second, and sixth author, and all interviews were conducted by the second author. The second author is a qualitative researcher, allied health clinician, with more than a decade of experience in primary care research. The second author coded the data and a subset of investigators formed an analysis team who met regularly (the first, second, third, fourth, and eighth author) to discuss findings.

In this sequential, explanatory mixed- methods study, the data are integrated during the interpretation, where the authors used the qualitative findings to explain and understand the quantitative time-series analysis.^21^

## RESULTS

Seven practices were recruited but one withdrew before commencement citing difficulties discussing alcohol use with patients via telehealth. The final results describe six intervention practices (one site provided quantitative data only, and another one, which was outside the PHN footprint, provided qualitative data only). All practices offered comprehensive general practice care and one had a strong clinical focus on alcohol and other drug use (Table 1).

**Table 1.** Characteristics of REACH project intervention clinics collected via a survey of the practice manager at baseline

**Clinic**	**Patient characteristics**					**Clinician characteristics**		
	**Regular patients, *n*^a^**	**Healthcare card, %^b^**	**Unemployed, %**	**Pension, %**	**Low-income household, %**	**Number of GPs (FTE)**	**Number of practice nurses (FTE)**	**Practitioner with interest in AOD?**
**1**	1050	>50	>50	>50	>50	1.6	1	Yes — one opioid maintenance therapy prescriber with an interest in alcohol dependence
**2**	8500	10–29	10–29	10–29	10–29	6	2.5	Yes
**3**	3000	<10	<10	10–29	Unsure	4	1	Yes — nurse AOD course
**4**	1500	>50	>50	>50	>50	1	1	Unsure
**5**	3057	>50	10–29	>50	>50	3.5	4	Yes — comorbid and complex cases
**6**	This sixth intervention site did not complete the pre-intervention survey or qualitative interviews; they did contribute quantitative data							

a *Regular patients — people seen at the practice on at least three occasions in the past 2 years.*

b *Healthcare card — marker of socioeconomic disadvantage as eligibility includes income test. AOD = alcohol and other drug. FTE = full-time equivalent. REACH = REducing AlCohol-related Harm.*

### Increased recording of alcohol histories

**Figure 1.**
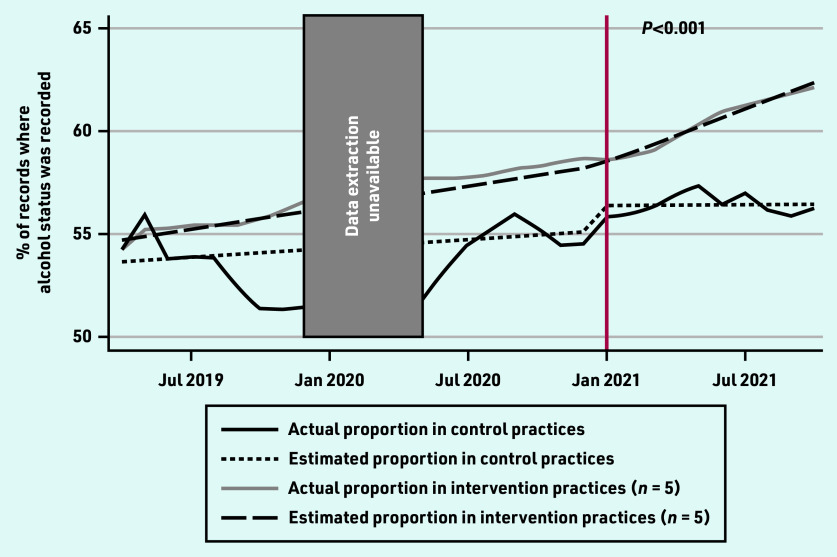
***Rates of alcohol status screening in control and intervention practices from routinely collected data in general practice. Intervention commenced in January 2021; see vertical red line. The*** **P*-value is for comparison of pre-and post-intervention slopes between the intervention practices and control practices.***

The non-intervention practices had a relatively constant rate of alcohol screening (Figure 1). For the period of January 2020 to April 2020 inclusive there was an error in the data extraction software.

Compared with their pre-intervention rates, the intervention practices significantly increased the rate at which they recorded patients’ alcohol status following the intervention (Table 2). More specifically, before the intervention, both intervention and non-intervention practices showed an increasing trend in the proportion of patient records where alcohol status was recorded. The slope of the post-intervention phase was steeper (indicating a faster increase in the proportion of filled records) in the intervention sites.

**Table 2.** Slope data for trends in alcohol status recording by intervention site status and intervention

**Variable**	**Alcohol status recorded (average per month), %**		**Pre-intervention slope (95% CI)**	**Post-intervention slope (95% CI)**	**Comparison of slopes, pre/post (95% CI)**
	**Pre-intervention, 21 months**	**Post-intervention, 10 months**			
**Non-intervention practices^a^**	55.2	56.4	1.002 (0.999 to 1.004)	1.000 (0.998 to 1.002)	0.998 (0.996 to 1.001)
**Intervention practices^b^**	56.7	60.4	1.004 (1.003 to 1.004)	1.007 (1.006 to 1.008)	1.004 (1.003 to 1.004)
**Comparison of control and intervention practices pre-/post-slopes**	—	—	—	—	1.006 (1.003 to 1.008)

a *The number of non-intervention practices varied per month, with an average of 344 and a range of 194–382 practices per month.*

b *One practice was outside the PHN catchment area so only five practices are included in the quantitative data. PHN = primary health network.*

### Factors that facilitated the increased use of brief interventions in primary care

Interviews were conducted with 19 participants (Table 3). Findings were organised across four themes:

- role of REACH resources in brief interventions (BIs);
- a whole-of-practice approach;
- practice factors: size, staff turnover, practice champion, and communication; and
- external facilitators and sustainability.

**Table 3.** Interview participants’ characteristics from the general practices and the PHN

**Practice/PHN interview**	**Position**	**Length of time in the practice/PHN**
**Practice 1**		
P1GP	GP	18 months (since opening)
P1PM	Practice manager	18 months
P1PN	Practice nurse	18 months

**Practice 2**		
P2GP	GP	4 years
P2PN	Practice nurse	6.5 years
P2CEO	Chief operating officer	7 months

**Practice 3**		
P3GP	GP	7 years
P3Rec	Receptionist	12 months

**Practice 4**		
P4GP	GP	23 years
P4PN	Practice nurse	9 years

**Practice 5**		
P5PM	Practice manager	3 years
P5CC	Care coordinator	26 years

**PHN**		
PHN1	Practice relationship manager	5 years
PHN2	Practice relationship manager	8 months
PHN3	Continuous quality improvement programme officer	10 months
PHN4	Project coordination	3 years
PHN5	Continuous quality improvement programme officer	6 years
PHN6	Continuous quality improvement programme officer	6 months
PHN7	Manager	6 years

*PHN = primary health network.*

### Role of REACH resources in BIs

There was a general understanding across both PHN and practice staff about the premise of the study and how to use the resources:

*‘It’s using tools to help discuss alcohol consumption with patients.’*

(Practice manager)

However, not all participants were familiar with the concept of BIs. Some clinicians seemed to misunderstand for what and whom BIs were best used as they talked about using them in the setting of alcohol dependence:

*‘… the REACH project is about reaching out to specific population and that is alcohol and drug, with drug and alcohol problems or drug and alcohol abuse.’*

(PHN)

Nonetheless, REACH resources were found to be acceptable to practices for both their visual appeal and availability in multiple languages, which was especially helpful for patients from diverse background or with low-literacy levels.

Practice staff and clinicians generally found the resources to be very helpful and, although not seen as entirely unique, they fit into existing practice routines:

*‘… one of the key reasons why we still signed up for it was that we didn’t have to change anything about the way that we ran; we can still talk about the REACH project in a normal consult … ’*

(Admin)

Their presentation and availability as a package were seen as valuable additions to preventive practices to raise awareness. Only two practices logged into the SMS/email portal and few sent any resources via the platform to patients. The added training, separate platform, and login plus the ongoing burden of the pandemic were barriers to its use.

### Whole-of-practice approach

Overall, a whole-of practice approach was advocated by the PHN:

*‘One of the things that we know was successful around these projects is having a whole-of-practice engagement around the project, and I think looking at the protocol, we set* [a] *project team as well as a project champion who then went back to the team. And unfortunately, that’s where some of it I think has also fallen down … one or two people from the practice team who knew about it and it was their responsibility to pass on some of that information, but if that didn’t happen and then that person left or that person moved to another role, then that continuity of the practice team still continuing with the activities and being clear on what they needed to do was lost.’*

(PHN)

In practices where only one person was involved in REACH, the demands of the pandemic and other stresses such as staff turnover, made their participation in REACH untenable. In practices with more than one person involved, the programme was better integrated into patient care. When more than one category of staff was involved, for example, practice nurses and managers, the practice was more likely to be more active in their involvement.

### Practice factors: communication, staff turnover, practice size, and practice champion

Practice size, staff turnover, communication flow, and the presence of a practice champion had an impact on implementation.

#### Lack of communication

A lack of communication about REACH across the practice was thought to be related to staff shortages or staff turnover:

Question: *‘*[do] *you have opportunities to reflect on REACH or discuss how it’s going? ’*

Answer: *‘I think we are — I don’t know what other practices are like, and I know it’s not an excuse, but we’re just very short on doctors, so there hasn’t been a second to even take time to meet the nurse to talk about it or any of that.’* (Receptionist)

#### Staff turnover

Staff turnover was an issue for practices where organisational memory was lost when key staff members left:

*‘I’ve only been here a year, so there might have already been something in place, but I think a lot of the clinical stuff around here isn’t communicated to management or to admin staff, I think they know what they’re doing, and they do it. I think in the past admin has not got involved in things like that.’*

(Receptionist)

#### Practice size

In smaller practices, communication seemed to be easier and less formal:

*‘Because it’s just me and him* [practice nurse and GP]*, if we need to discuss something, either I go into his room or he comes into my room, and we just discuss it.’*

(Practice nurse)

#### Practice champion

Practices that used a practice manager as the practice champion seemed to have more formal and regular communication across the practice, making REACH more accessible and supporting practice-wide involvement:

*‘… we include REACH in any of the meetings. Me, in particular, with my meeting with my nurses — or sometimes all I have to do is I’d go out to my reception and say, “How are we doing with the survey? Make sure everyone is provided the survey, and ask them if they have questions. They can talk to the nurses and all.” So we come up with that kind of — you know, a very simple but so far effective way of making sure that we’re integrating REACH project information into our daily clinical operation.’*

(Practice manager)

In two practices, GPs were the practice champion and it was not comprehensively implemented in either practice. In one practice, the GP was overwhelmed by the pandemic and did not implement REACH. In the other practice, the GP felt unsupported and, as she stated, ‘on my own’ in implementing REACH. The involvement of the practice champion was a key facilitator for implementation.

### External facilitators and sustainability

External facilitators included regular communication with the PHN including reporting and key performance indicators, particularly in relation to PIPQI.

The regular meetings between the practices and their PHN relationship manager were an essential engagement tool to communicate data recording with practices:

*‘And we went through that and data — showing practices or clinicians data is like — they love it. They love it. They want to know where they can improve.’*

(PHN)

Six relationship managers in the PHN had one practice each to work with on REACH. However, because of staff turnover, miscommunication, and pandemic complications, engagement meetings did not occur as often as were intended.

Further, both PHN and general practice participants identified that REACH aligned with the PIPQI incentive. Practice staff were aware of their data needs for PIPQI, and REACH provided a platform for engagement with that system and feedback from the PHN:

*‘*[The software for data extraction provided by the PHN] *helps us a lot because it helps us monitor how we’re progressing and our performance in doing data cleansing, in updating our record for indexes for PIPQI, which includes alcohol level and smoking level.’*

(Practice manager)

The PHN staff also recognised the link between REACH and the national PHN key performance indicators that are set by the Australian Government allowing the seamless involvement of the PHN in implementation:^19^

*‘… we were just interested in providing a project like that to the general practices in our region, as the objective of the project was, it aligned well with what we were doing and the, the topics that we focus on like, disease prevention or systems improvements … ’*

(PHN)

## DISCUSSION

### Summary

This study designed a programme to increase ABIs within general practices, assessed its effectiveness, and identified factors that made its implementation more successful. The REACH programme resulted in small but significant increases in the rate of recording of alcohol status in practice sites. Although small, this increase occurred in the challenging context of service delivery during the COVID-19 pandemic. Small changes delivered at scale may deliver measurable health gains if delivered at the population level through a low-intensity intervention. As this study was undertaken during COVID-19 with major service upheaval, the effect size is likely to bias towards an underestimate.

PHNs were critical for success as they facilitated practice meetings and provided copies of resources and real- time practice- level data. Some clinicians will require new knowledge and upskilling to ensure they are appropriately and effectively using BIs that could be facilitated by PHNs. Clinicians reported that REACH resources were warmly received because they were visually appealing, available in relevant local languages, and provided in both hard copy and electronic formats. It was problematic that the SMS/email portal required a separate login and training. The authors of this study recommend that resources are refreshed annually to ensure the REACH programme remains salient.^33^

### Strengths and limitations

The strengths of this study include the theory- informed approach to implementation allowing the development of a strategy to increase the likelihood of ABI uptake. The qualitative data gave a richer picture of the process of implementation than only quantitative data. Non-intervention and intervention practices did not have the same pre-intervention levels and behaviour, and, although all practices had initial alcohol recording below expected national standards, recruited practices may be more interested in preventive health care. The recording of alcohol history is a surrogate marker for BIs; however, alcohol screening is an established proxy for ABI delivery that has been used in other primary care studies.^13^^,^^15^ Also, it is unlikely that a clinician would take a history of alcohol use without providing further information if indicated. As the authors used routinely collected data, information on the percentage of patients seen and screened during the trial was not available. The comparatively small number of practices in the intervention group may mean that there was a reduced sensitivity in detecting differences in practice behaviour. Patient-level data could not be examined and no characteristics such as gender were available at the population level. Evidence for the digital divide as a social determinant of health is mounting, and the online approach that was necessary because of COVID-19 may have disadvantaged some patients.^38^

### Comparison with existing literature

Studies on financial incentives in the UK have clearly demonstrated that a comprehensive approach, including a focus on workflows and clinician skillset for ABIs,^13^^–^^15^ is needed to increase the delivery of ABIs in primary care. Research from both Norway^39^ and Australia^40^^,^^41^ highlight the importance of the context of the consultation (for example, seemingly unrelated to alcohol) for ABIs, adding to the literature on the need for system support for clinicians to undertake screening for substance misuse in a primary care setting.^42^ Focusing solely on clinician deficiencies in knowledge, attitude, time, and resources is not likely to be effective because of the broader and enmeshed social and cultural dimensions of alcohol drinking, including within the patient– doctor relationship.^43^ There are issues around stigma, shame, and identity that must be navigated in longitudinal patient–doctor relationships in primary care.^44^ In the context of the REACH programme, it is perhaps the visible and explicit normalisation of ABIs for both patients and doctors that creates the environment where ABI implementations are more likely to be seen as acceptable and sustainable.

The results of the current study indicate that identifying a ‘practice champion’ and at least two categories of team members seems to assist with a whole-of-practice approach. There are other areas of primary care implementation that have shown similar findings, specifically around a whole- of-practice approach and engaging with PHNs.^45^^–^^47^ A whole-of-practice approach recognises the effectiveness of engaging all practice staff and considering practice context and capacity to achieve practice improvement.^48^ Genuine support for implementation across a region has also been described as essential in nutrition interventions in general practice, where single clinics or practitioners may not elicit meaningful changes without broader support.^49^

Factors associated with successful implementation of teamwork interventions include local contextual factors and external factors. Local factors such as: size, the percentage of active clinicians in the practice involved in the intervention, power dynamics, leadership, and the physical environment of the practice^45^^,^^46^ may have an impact on successful implementation. The current study similarly indicates the importance of the percentage of active clinicians involved, as, where only one clinician was engaged in implementation, REACH was less likely to be used. Some studies show that the external contextual factors that may have an impact on the success of teamwork interventions include: funding, the approach taken to team-based care by professional organisations, the degree of accountability required by practices, and how linked practices are to their broader community.^47^ External factors also played a role in the implementation of REACH: alignment with the requirements of PIPQI and the degree to which practices were connected to their PHN both influenced implementation.

The authors’ prior work to inform the REACH programme found that there were barriers and facilitators to the uptake of ABIs at multiple levels of the system, from the individual patient and clinician, through to community norms about alcohol consumption.^45^ Although there were a range of influences supporting the implementation of REACH, more practical barriers such as time constraints and staff turnover still need to be addressed. The REACH programme does not address community and public health messaging and this could be a mechanism that further enhances patient knowledge about alcohol harms.

### Implications for research and practice

It is possible to increase the routine recording of alcohol status in electronic medical records using the REACH programme, demonstrating the opportunities for primary care to deliver ABIs with appropriate support and infrastructure. The REACH programme included resources aimed at patients, clinicians, and practices to support implementation and increase discussions about alcohol in primary care. The clinic-level real-data feedback loop to practices was a critical element for implementation success. Evidence on salience shows that resources need to be updated and renewed over time to ensure ongoing interest, as novelty is one aspect of the resources that will draw participant attention back to them over time.^26^ The authors of the current study recommend that resources are refreshed annually to ensure continued relevance and salience.

The current policy alignment in Australia between PHN key priority areas, the PIPQI, and community needs demonstrates how policy can support the uptake of BIs in primary care. This policy alignment may be of interest to other nations, particularly the UK and Canada, which have similar primary care commissioning organisations to PHNs. The increased uptake of BIs is supported at multiple policy levels, from federal policies identifying priority areas for PHNs as well as the current PIPQI that encourages general practices to report their alcohol recording data. These outer policy-level factors are then reinforced by REACH by having a practice champion and augmented by involving more than one person in the practice and ensuring clinicians are included.

## Data

Northern West Melbourne Primary Health Network are the data custodians and requests for access to the quantitative data will need to be approved by them. The qualitative data are securely stored and are available after appropriate ethics approval.
